# Analysis of the neurotoxic effects of neuropathic organophosphorus compounds in adult zebrafish

**DOI:** 10.1038/s41598-018-22977-4

**Published:** 2018-03-19

**Authors:** Melissa Faria, Inmaculada Fuertes, Eva Prats, Jose Luis Abad, Francesc Padrós, Cristian Gomez-Canela, Josefina Casas, Jorge Estevez, Eugenio Vilanova, Benjamin Piña, Demetrio Raldúa

**Affiliations:** 1 Department of Environmental Chemistry, Institute of Environmental Assessment and Water Research (IDAEA-CSIC), Jordi Girona 18, E-08034 Barcelona, Spain; 2 CID-CSIC, Jordi Girona 18, E-08034 Barcelona, Spain; 3 Department of Biomedicinal Chemistry, Institute for Advanced Chemistry of Catalonia, (IQAC-CSIC), Jordi Girona 18, E-08034 Barcelona, Spain; 4 Fish Diseases Diagnostic Service, Facultat de Veterinaria Universitat Autònoma de Barcelona, 08190 Bellaterra (Cerdanyola del Vallès), Spain; 5 Institute of Bioengineering, University “Miguel Hernandez” of Elche, Alicante, Spain

## Abstract

Inhibition and aging of neuropathy target esterase (NTE) by exposure to neuropathic organophosphorus compounds (OPs) can result in OP-induced delayed neuropathy (OPIDN). In the present study we aimed to build a model of OPIDN in adult zebrafish. First, inhibition and aging of zebrafish NTE activity were characterized in the brain by using the prototypic neuropathic compounds cresyl saligenin phosphate (CBDP) and diisopropylphosphorofluoridate (DFP). Our results show that, as in other animal models, zebrafish NTE is inhibited and aged by both neuropathic OPs. Then, a neuropathic concentration inhibiting NTE activity by at least 70% for at least 24 h was selected for each compound to analyze changes in phosphatidylcholines (PCs), lysophosphatidylcholines (LPCs) and glycerolphosphocholine (GPC) profiles. In spite to the strong inhibition of the NTE activity found for both compounds, only a mild increase in the LPCs level was found after 48 h of the exposure to DFP, and no effect were observed by CBDP. Moreover, histopathological evaluation and motor function outcome analyses failed to find any neurological abnormalities in the exposed fish. Thus, our results strongly suggest that zebrafish is not a suitable species for the development of an experimental model of human OPIDN.

## Introduction

Organophosphorus compounds (OPs) are used in agriculture and industry and also include some chemical warfare agents. Although most OPs exhibit acute toxicity due to the inhibition of acetylcholinesterase (AChE), only neuropathic OPs result in OP-induced delayed neuropathy (OPIDN), a chronic neurodegenerative disorder characterized by a central-peripheral distal axonopathy of the longest sensorimotor axons^1^. OPIDN becomes apparent 1–4 weeks after exposure to a neuropathic OP, and the clinical course include paresthesia in the distal extremities, sensory loss, ataxia, flaccid paralysis and, finally, spastic paralysis^2^. More than 70,000 human cases of OPIDN have been identified in the last 75 years^3^, and in most of them, the neuropathic OP responsible was tri-ortho-cresyl phosphate (TOCP), a chemical used for many different industrial applications, including as a plasticizer, flame retardant, and antiwear additive in lubricants and hydraulic fluids. Although humans are highly susceptible to OPIDN, not all animal species are sensitive. Susceptible species, including cows, sheep, water buffalos, dogs, cats, and chickens, are generally larger and have longer axons than those with low sensitivity, such as rodents^1^.

The toxicological mode of action of neuropathic OPs resulting in OPIDN has been debated for many years and is still unclear. The most accepted view is that inhibition and aging of neuropathic target esterase (NTE), the protein product of the patatin-like phospholipase domain containing 6 (*PNPLA6*), is the molecular initiating event triggering the adverse outcome pathway resulting in OPIDN^4,5^. In fact, in susceptible species, a single dose of neuropathic OP inhibiting >70% NTE activity for at least 24 h predictably results in clinical signs of neuropathy after 1–3 weeks^6,7^, and this process can be measured using the artificial substrate phenyl valerate (PV)^8^. NTE is an enzyme anchored to the cytoplasmatic face of the endoplasmic reticulum (ER) with lysophospholipase (lysoPLA) and phospholipase B (PLB) activities, catalyzing under physiological conditions the deacylation of phosphatidylcholines (PCs) and lysophosphatidylcholines (LPCs) to glycerolphosphocholine (GPC)^9–11^. Thus, inhibition and aging of NTE by neuropathic OPs will result in the irreversible inactivation of its phospholipase activities, although the final effect on the brain PCs and LPCs homeostasis is contradictory^12–15^. In fact, the hypothesis in which the loss of the catalytic function of NTE is the molecular initiating event for OPIDN development has not been demonstrated and alternative hypothesis for the role of NTE have been proposed, including the impairment of a non-catalytic function^16,17^ or a toxic gain of function of this protein^2,18,19^. Although there are no doubts about the implication of NTE in the development of OPIDN, there are uncertainty either in the chain of molecular/cellular events after the primary event and also the potential role of many other protein/esterases also chemically modified in the early stages. Short after the NTE inhibition other molecular alterations are follows, including the activation of Ca2+/calmodulin kinase II (CaMKII), the consequent hyperphosphorilation of axonal microtubules and neurophilaments, and the resulting alteration of axonal retrograde transport^20–23^. Moreover, Ca^2+^ dysregulation and altered proteases have been also described^24,25^. Finally, as neuropathic and non-neuropathic OPs can interact with multiple esterases in nerve tissue, apart from NTE, therefore, the potential role of other esterase or esterase-like proteins with or without known catalytic properties, cannot be discarded^26,27^.

Although different pharmacological agents have been proposed for prophylaxis/treatment of human OPIDN^3,25^, there is not yet a single specific treatment available for clinical practice to prevent or alleviate the effects of severe OPIDN. Understanding the mechanisms of toxicity of neuropathic OPs resulting in OPIDN should be critical for identifying potential therapeutic targets and the further development of specific therapies. Adult hen has been the preferred animal model for analyzing the toxicity pathways associated to OPIDN development, as this model develops the histological lesions and clinical signs, such as ataxia and paralysis, in predictable manner after single dose of neuropathic OPs and it is the model recommended for testing in regulatory process^28,29^, although clinical effect can be showed in rats under potentiating conditions^30^. However, not many laboratories are in condition for testing with chickens, and no definitive advances have been done in the identification of the adverse outcome pathways involved in OPIDN over the last few decades by using chemical models of human OPIDN developed in hens and rodents. *In vitro* models have been proposed based on several endpoints, for example in neurite outgrowth^31^ or just on esterase inhibition in neural cell lines^32,33^ or neurotransmitter release^34–36^. However, the need of testing in animal model are still needed for assessing neurotoxicity and neurodevelopmental toxicity in a integrated approach^33^. Thus, the suitability of new animal models of human OPIDN for studying the pathophysiological mechanisms and for screening chemicals against the identified therapeutic targets should be explored.

Zebrafish (*Danio rerio*) is a vertebrate species, with a similar overall nervous system organization to humans, and is increasingly used to model human diseases, including sensory-motor distal axonopathies and other motor neuron diseases^37–40^. The zebrafish model presents many advantages for neurosciences research, including that is one of the few vertebrate models suitable for high-throughput screening of small molecules libraries, and there is available an extensive collection of useful resources, such as genetic and behavioural tools (reviewed at Babin *et al*.^37^). Interestingly, an *in vivo* zebrafish model for upper motor neuron axon degeneration, based on axotomy of the Mauthner cell, has been recently proposed^41,42^. Suitable models of early- and late-onset pure hereditary spastic paraplegia (HSP), characterized by the distal axonopathy of the longest axons of upper motor neurons, have been built in zebrafish embryos during the last years. Finally, the presence of transcripts of *pnpla6*, the gene encoding NTE, has been recently reported in zebrafish embryos, and knockdown of this protein early in development resulted in motor neuron defects^43^. This support that zebrafish might be a potential model for testing neurotoxicity induced by neuropathic OPs.

The expression of NTE in differentiating embryos^44^ and the role of NTE in the embryonic development was demonstrated in NTE deficient mices^45,46^ and it was demonstrated a role of the NTE protein and its encoding gene in the differentiation^47^, associated to alteration of in the pathways related with lipid membrane formation and tube morphogenesis^44,48^. As zebrafish is also considered a suitable non-mammalian animal model for testing development, and moreover it may be also extrapolated for assessing neurodevelopmental toxicity as it has been included in proposals for integrated assessment of developmental toxicity from *in vitro* to *in vivo* extrapolation approaches^33^. This also support that it is of interest exploring the possibilities of this fish model.

In the present study, we aimed to assess the effects on adult zebrafish by two different neuropathic OPs (CBDP and DFP), causing OPIDN in humans. Inhibition and aging of brain PV-NTE activity, phospholipid (PCs, LPCs, and GPC) profiles, histopathological effects, as well as effects on the motor function outcome have been characterized.

## Results

### PV-NTE activity in adult zebrafish brain

**Figure 1:**
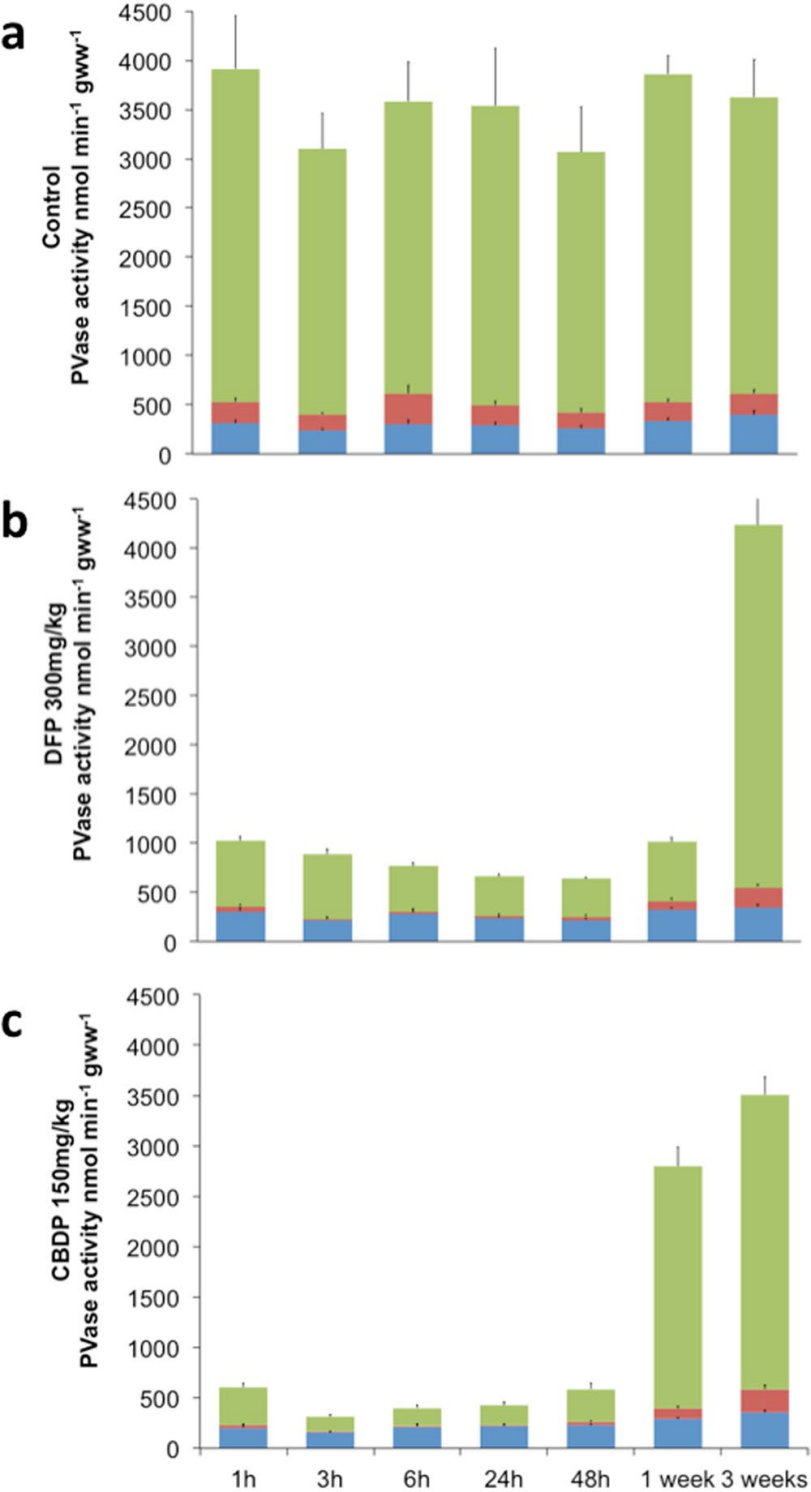
Representations of proportional time dependent inhibition of PVase components by DFP and CBDP. It is showed the total PVase activity (total bars) and the following components: paraoxon-sensitive activity (green), paraoxon-resistant, mipafox-sensitive (NTE, red) and (paraoxon + mipafox)-resistant activities (blue). Panels: activities from control (**a**), 300 mg/kg bw DFP (**b**) and 150 mg/kg bw CBDP exposed zebrafish (**c**). The total average PVase activity of control fish was 3527.3 ± 486 nmol min^−1^ gww^−1^ (100%) distributed as, 3012.9 ± 415 (85.4%), 211.5 ± 38 (6%) and 302.9 ± 32 (8.6%) nmol min^−1^ gww^−1^ for the paraoxon-sensitive, NTE (paraoxon-resistant and mipafox-sensitive), and (paraoxon + mipafox)-resistant, respectively.

Although concentrations of neuropathic OPs inducing ≥70% inhibition of PV-NTE activity in the central and peripheral nervous system are known to produce delayed neuropathy in many different animal models, there is no information about the potential adverse effect of these compounds in fish. The results of this paper show that the average total PVase activity in zebrafish is 3527.2 ± 486.3 nmol min^−1^ gww^−1^ for the total PVase activity, while it is 514.4 ± 62.0 for the paraoxon resistant activity (14.6% of total PVase activity) and 302.9 ± 32.6 nmol min^−1^ gww^−1^ for the paraoxon + mipafox resistant activity (8.6% total PVase activity), and therefore the PV-NTE activity (paraoxon-resistant, mipafox sensitive) is around 211.5 ± 38.4 (6.0% of total PVase activity; Fig. 1 and Table S1).

### Inhibition and aging of zebrafish NTE after a single dose of neuropathic OPs

**Figure 2:**
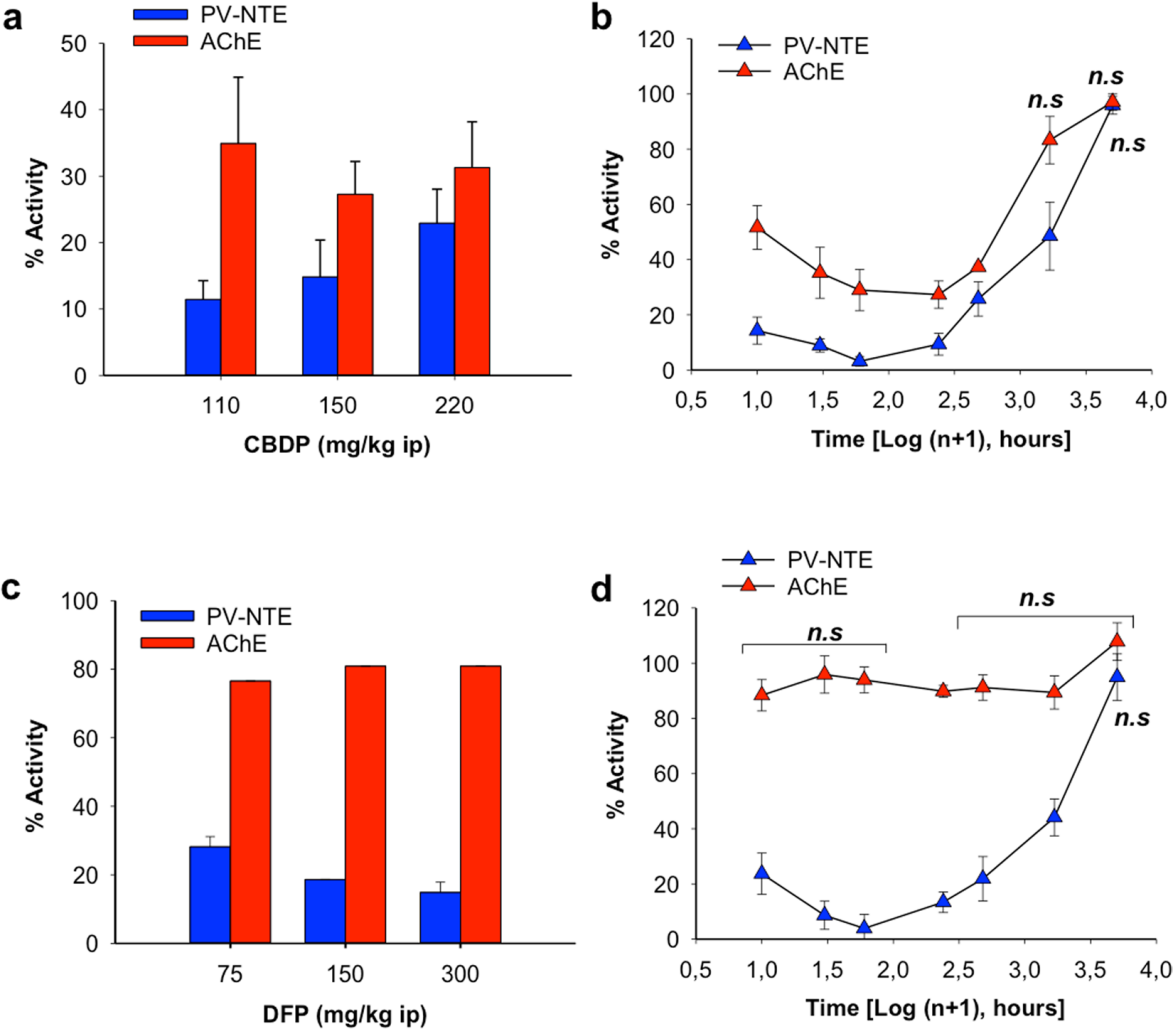
Dose- and time-dependent inhibition of zebrafish brain PV-NTE and AChE activities by neuropathic OPs. (**a**) Inhibition of PV-NTE (blue bars) and AChE (red bars) activities in the zebrafish brain 24 h after exposure to 110, 150 and 220 mg/kg. (**b**) Time-course of the inhibition of PV-NTE (blue triangles) and AChE (red triangles) activities in the zebrafish brain after a single dose of 150 mg/kg CBDP. (**c**) Inhibition of zebrafish PV-NTE (blue bars) and AChE (red bars) activities in the zebrafish brain 24 h after exposure to 75, 150 and 300 mg/kg of DFP. (**d**) Time-course of the inhibition of PV-NTE (blue triangles) and AChE (red triangles) activities in the zebrafish brain after a single dose of 300 mg/kg DFP. All PV-NTE and AChE activities are shown as the percentage (mean ± SEM) relative to the control values. Average fish brain PV-NTE and AChE activities from control individuals were 211.5 ± 38.4 nmoles min^−1^ g wet weight^−1^ (n = 56) and 210.12 ± 4.62 nmoles min^−1^ mg^−1^ (n = 36), respectively. Responses were compared to their respective controls with two-tailed Student’s *t*-tests. Significant difference was set at p < 0.05. All data, with the exception of those indicated with *n.s*. (not significant), were significantly different from the controls.

The hypothesis that these chemicals also inhibit NTE activity in adult fish brain was tested using male adult zebrafish as an experimental model and selecting CBDP and DFP as prototypic neuropathic OPs. The dose-response analysis of the effects of CBDP on mortality (Supplemental Fig. 1) showed a 24 h maximum tolerated dose (MTD) for mortality of 150 mg/kg body weight (bw) CBDP. Then, a dose-response analysis was performed, assessing the PV-NTE activity in the brains of fish exposed for 24 h to CBDP doses ranging from 110 to 220 mg/kg bw. The results showed that PV-NTE activity in the brains of fish exposed to this range of concentrations was inhibited 71–92% (Fig. 2a). As neuropathic OPs must inhibit NTE ≥ 70% for at least 24 h to induce delayed neuropathy in susceptible species, the time-course inhibition of both PV-NTE and AChE activities induced by 150 mg/kg bw CBDP (MTD) was analyzed (Fig. 2b, and Table S2). PV-NTE inhibition in brain samples of CBDP-exposed fish remained over 70% from 1 to 48 h after exposure. One week after exposure, inhibition levels were still high, and PV-NTE activity returned to the control levels three weeks after exposure. CBDP, during the first 48 h after-exposure, also affected the paraoxon sensitive and paraoxon + mipafox resistant components of PVase activity, which were reduced by 91.75% and 27%, respectively, compared to control values of the same time period (Fig. 1 and Table S1). The lowest PV-NTE activity during the whole time-course was observed 6 h after CBDP exposure (3.55 ± 3.56%). Although CBDP also inhibited AChE activity the inhibitory effect of this chemical was more potent towards PV-NTE than AChE in zebrafish. Thus, one hour after exposure, PV-NTE activity was already as low as 11.49 ± 6.39% relative to 57.93 ± 15.41% of AChE activity. AChE activity decreased in a time-dependent manner for approximately 24 h, after which it began to gradually increase and fully recovered 1 week after CBDP exposure (p = 0.293).

The second selected compound, DFP, was tested at a concentration range of 2.4 to 300 mg/kg bw without inducing mortality (n = 24, two independent experiments; see Supplemental Fig. 1). Since no mortality was observed across the DFP doses tested, PV-NTE and AChE activities were measured with all doses except the lowest. The results showed that both enzymes were inhibited in a dose-dependent manner (Supplemental Fig. 2). The three highest DFP doses tested inhibited PV-NTE activity by more than 70% (Fig. 2c), and 300 mg/kg bw DFP was the dose selected for the time-course experiments. PV-NTE activity decreased to 23.7 ± 7.48% of the control values as early as 1 h after DFP exposure, reaching a plateau of lowest activity (3.87–13.39% of the control values) between 3 and 24 h (Fig. 2d and Table S2). Then, similarly to CBDP, PV-NTE activity levels were recovered 3 weeks after exposure. DFP’s also affected the activity of PVase paraoxon sensitive component, which was decreased by 83%, and did not affect the paraoxon + mipafox resistant component (p > 0.05) (Fig. 1, Table S2). Interestingly, although the brain AChE activity across time in the fish treated with DFP was always slightly lower than that of control fish, these differences were not significant, with the exception of the 24 h sampling.

Although the results presented above demonstrate that CBDP and DFP inhibit PV-NTE activity in adult zebrafish brain, it is the presence of organophosphorilated and aged NTE rather than the absence of its catalytic activity that leads to OPIDN^7^. Whereas these compounds induce inhibition and aging of NTE in different animal models, no information is currently available on their aging potential for zebrafish NTE. Thus, the capability of fluoride ion to reactivate NTE from zebrafish brain previously inhibited across time by neuropathic OPs has been explored as an *in vivo* marker for aging, where less reactivation corresponds to higher NTE aging. Since the majority of NTE aging is known to occur within 24 h of exposure^49^, time course aging assessment was carried out within the first 24 h post exposure period. As Table 1 shows, CBDP and DFP aged zebrafish NTE, although with some differences in the time-course. Across time exposure, CBDP and DFP inhibited neurotoxic esterases response to KF reactivation was strongest 1 hour after exposure. Nevertheless, at this time point, enzyme reactivation in CDBP exposed fish was very mild when compared to DFP, 25.7 vs. 60.02%, respectively. Within the following 6 to 12 h after exposure, response to the reactivation agent essentially decreased for both OPs, suggesting the onset of aging. The decrease was more dramatic for DPF, but CDBP reached the lowest reactivation capacity at 6 h post-exposure (12.62%). 24 h after exposure, reactivation of NTE by KF was more efficient, especially for DFP.

**Table 1:** Reactivation of PV-NTE activity in zebrafish brain tissue inhibited by the neurotoxic OPs CBDP and DFP.

Inhibitor	Time (hours)	%NTE		%Reactivation
		KCl (±SEM)	KF (±SEM)	
CBDP (150 mg/kg)	1	8.46 ± 2.2	36.40 ± 10.8	25.77^ab^
	6	8.29 ± 2.3	16.54 ± 5.8	12.62^a^
	12	6.39 ± 3.3	20.76 ± 4.4	13.86^a^
	24	−0.49 ± 3.7	19.76 ± 9.1	21.53^a^
DPF (300 mg/kg)	1	12.43 ± 3.2	74.98 ± 8.7	60.02^b^
	6	8.79 ± 1.3	26.77 ± 5.6	19.01^c^
	12	6.85 ± 2.0	21.71 ± 4.7	14.45^c^
	24	5.13 ± 2.0	36.15 ± 4.6	35.04^b^

Fish were exposed, via i.p., to 150 and 300 mg/kg of CBDP and DFP, respectively. Brain tissue samples where taken 1, 6, 12 and 24 h after exposure and processed for reactivation measurement. Results are from two independent experiments (n = 4 per condition) expressed in % relative control and are presented as mean ± SEM. Reactivation was calculated as follows: 100 × [(% NTE_KF_ − % NTE_KCl_)/(100 − % NTE_KCl_)]. Different letters indicate significant differences (p < 0.05) between responses (OPs and time periods) following one way ANOVA and Tukey post hoc comparison test.

### Lipidomic analysis

**Figure 3:**
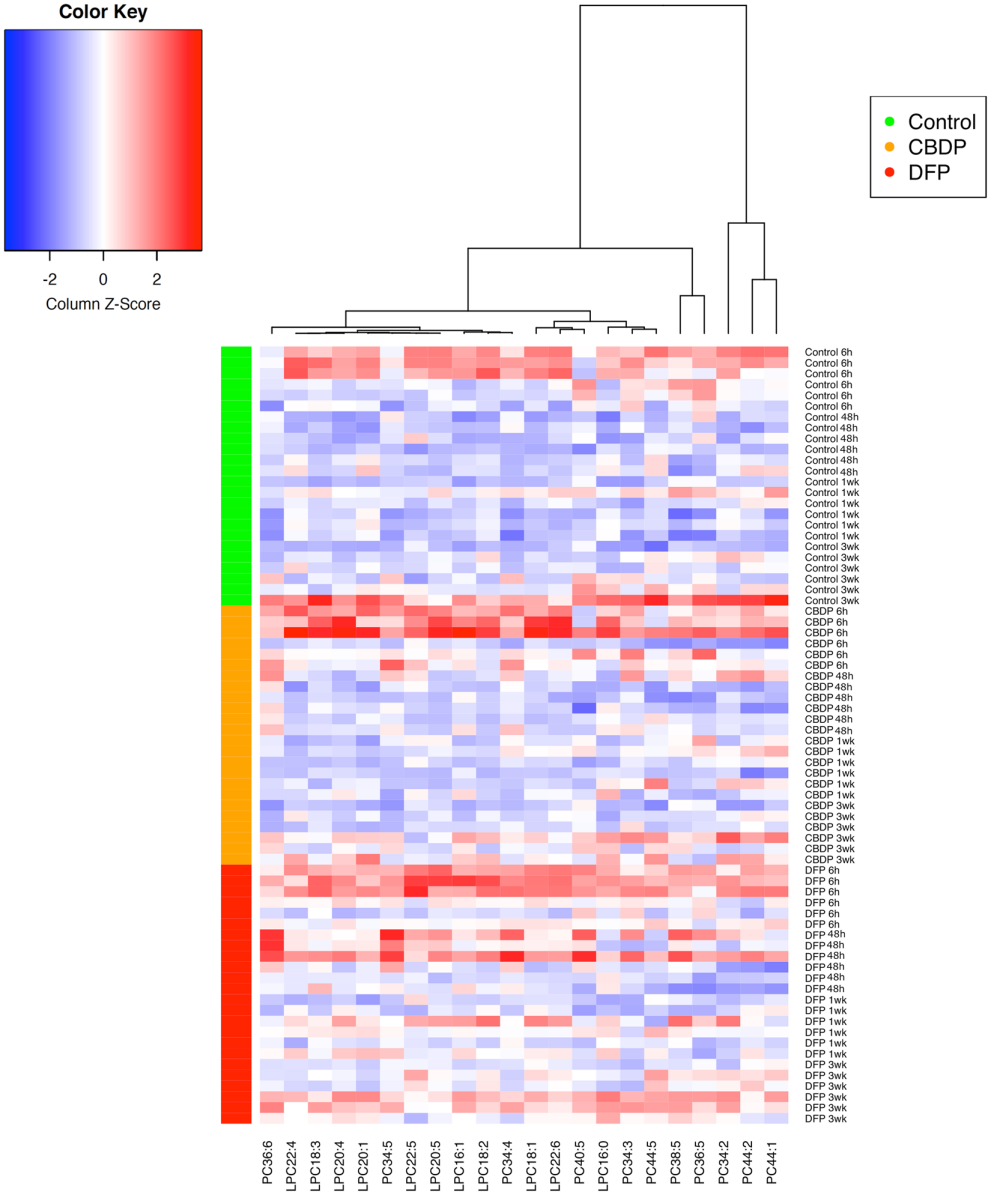
Heatmap of phospholipid variations in treated and untreated samples along the observed period (6h-3wks). Only lipids with significant variations (two-ways ANOVA, Table 2) are displayed. Note that clusterization did not separate PCs from LPCs. Colors in the left indicate the different treatments (green for controls, orange for CBDP and red for DFP).

Once determined the strong inhibitory effect and aging of the neuropathic compounds CBDP and DFP on the PV-NTE activity in the brain of adult zebrafish, their effect on the phospholipase activity of this enzyme was evaluated. First of all, we analyzed any potential effect of the selected vehicle, corn oil, on the phospholipidomic profile in the brain of the injected fish. Thus, when the brain levels of PCs, LPCs or GPC in non-injected fish and vehicle-injected fish 6, 12 and 24 h after injection were compared, no significant differences (p > 0.05) were found for any of the 57 selected phospholipids (data not shown). Then, the profiles of PCs, LPCs and GPC were analyzed in the brain of control, CBDP- and DFP-treated zebrafish 6 h, 48 h, 1 week and 3 weeks after injection (Supplemental Dataset 1). In spite of the effect observed on PV-NTE activity, the total phospholipid content did not show significant changes during the observed period, with or without treatment (two-way ANOVA, p > 0.05, data not shown). Analysis of individual compounds revealed that 22 lipids (12 PCs and 10 LPCs) show temporal variations, and seven of them respond to the at least one of the treatment, with a low interaction between the two variables (two-way ANOVA, p < 0.05, Table 2). These figures changed to 16 and 2, respectively, after applying the Benjamini-Hochberg post-hoc analysis (Table 2), indicating that the effect of the treatments was very weak, if any. The pattern of temporal changes for all affected lipids, with a maximum at 6 h after injection and a progressive reduction with time, with some weaker increase in the older animals. This pattern was very similar for PCs and LPCs (Fig. 3, quantitative analysis in Supplemental Fig. 3), demonstrating no alterations in the balance between PCs and LPCs linked to the treatments.

**Table 2:** Results from the two-way ANOVA (Treatment × Time) of phospholipid concentrations. Only compounds showing significant variations are included.

Lipid	*p*-values^a^			*p*-values (FDR correction)^b^		
	Treatment	Time	Treatment*Time	Treatment.fdr	Time.fdr	Treatment*Time.fdr
PC34:2	n.s.	3.35E-02	n.s.	n.s.	n.s.	n.s.
PC34:3	n.s.	8.70E-05	n.s.	n.s.	0.001044	n.s.
PC34:4	0.004009	1.19E-03	n.s.	n.s.	0.006616	n.s.
PC34:5	0.000516	1.86E-03	0.0419	0.0186	0.009581	n.s.
PC36:5	n.s.	3.80E-04	n.s.	n.s.	0.003416	n.s.
PC36:6	0.00041	3.28E-04	n.s.	0.0186	0.003374	n.s.
PC38:5	0.034923	2.37E-02	n.s.	n.s.	n.s.	n.s.
PC40:5	n.s.	6.13E-03	n.s.	n.s.	0.029442	n.s.
PC44:1	n.s.	1.41E-02	n.s.	n.s.	n.s.	n.s.
PC44:2	n.s.	3.80E-02	n.s.	n.s.	n.s.	n.s.
PC44:5	n.s.	3.31E-02	n.s.	n.s.	n.s.	n.s.
LPC16:0	n.s.	2.48E-02	n.s.	n.s.	n.s.	n.s.
LPC16:1	0.021848	8.29E-04	n.s.	n.s.	0.004974	n.s.
LPC18:1	0.011645	7.43E-06	n.s.	n.s.	0.000268	n.s.
LPC18:2	n.s.	4.90E-04	n.s.	n.s.	0.003465	n.s.
LPC18:3	n.s.	7.29E-05	n.s.	n.s.	0.001044	n.s.
LPC20:1	n.s.	8.71E-03	n.s.	n.s.	0.039203	n.s.
LPC20:4	0.028982	5.29E-04	n.s.	n.s.	0.003465	n.s.
LPC20:5	0.032881	9.85E-07	n.s.	n.s.	0.000071	n.s.
LPC22:4	n.s.	4.51E-04	n.s.	n.s.	0.003465	n.s.
LPC22:5	0.042325	6.45E-05	n.s.	n.s.	0.001044	n.s.
LPC22:6	n.s.	2.51E-05	n.s.	n.s.	0.000601	n.s.

^a^p-value for null hypothesis, uncorrected two-way ANOVA Treatment × Time.

^b^p-values corrected for multiple testing (false discovery rate, fdr).

ASCA results (Table S3) indicated that a dominant part of variation in the lipidomic data is coming from residual (non-factor) variability (residuals ~78%) and another part is coming from the applied factors (≤21.2%). No effects were observed for treatment and exposure time factor which were statistically assessed from the results of the permutation test (Table S3, p ≥ 0.05). On the other hand, results of the permutation test also confirmed that the interaction of these two factors was not significant (p > 0.05). PCA scores of the first and second component for the “treatment” factor (Supplemental Fig. 4a) explained 68.40% and 31.60%, respectively, of the variation observed for this factor. On the other hand, scores for the “exposure time” factor data matrix (Supplemental Fig. 4b) displaying the PC1 (with 90.24% of the total data variance) in front of the PC2 (with 7.42% of the total data variance). Finally, PC1 scores of the interaction matrix did not show any specific pattern, as there was no systematic increasing or decreasing trend in the sample scores at the different treatments respect to exposed times (Table S2). These results of the ASCA analysis indicated that CBDP and DFP did not induced significant changes in the lipidomic profile at any of the selected times.

### Histopathological analysis

**Figure 4:**
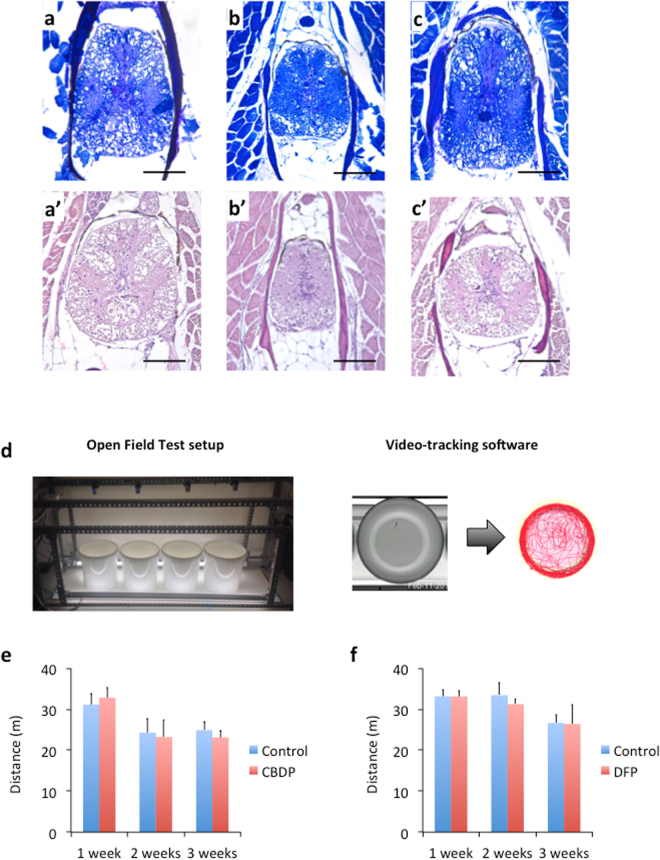
Neither histopathological changes (**a–c**) nor differences in the motor function outcome in the open field paradigm (**d–f**) were found after a single dose of CBDP or DFP. (**a–c**) Representative sections of the caudal spinal cord sections of adult zebrafish from the control (**a,a’**), CBDP (**b,b’**) and DFP (**c,c’**) groups, 3 weeks after injection. Sections were stained with Luxols Fast Blue (**a–c**) and Hematoxylin & Eosine (**a’–c’**); (**D**) The OFT setup allowed us to simultaneously analyze four adult fish, video-recording and tracking the movement of the fish for 6 min. (**e,f**) No significant differences between the total distance traveled by the control and the CBDP (**b**) and DFP (**c**) groups were found at any time. Data from 2–3 independent experiments are shown.

No histopathological alterations or changes were detected in any of examined sections from the fish at 1, 2 or 3 weeks after exposure to CBDP or DFP. The morphology of the spinal cord and the histological structures in the sections was similar in the exposed fish as in the control fish (Fig. 4a–c).

### Locomotor behavior remains unaltered after a single dose of CBDP and DFP

The OFT paradigm was used for testing the motor function outcome, using the total distance traveled as an endpoint reflecting general motor/neurological phenotypes (Fig. 4d). Before determining the effect of neuropathic OPs on the OFT behavior, we examined the potential confounding effect of circadian rhythms and habituation on this experimental paradigm. First, our results showed that the most stable period during the day to perform the experiments was from 09:00 to 13:00 h (Supplemental Fig. 5a). Moreover, a clear effect of habituation after repeated measures was found (Supplemental Fig. 5b). As a result, all the OFT studies were performed between 09:00 to 13:00 h, using fish experimentally naïve to avoid the inter-sessions habituation response.

When the locomotor behavior of adult fish 1–3 weeks after a single ip dose of carrier (control), 150 mg/kg CBDP or 300 mg/kg DFP was tested in the open field paradigm, no significant differences were found in the total distance traveled between the control and the two treated groups at any selected time (Fig. 4e–f).

## Discussion

The potential of adult zebrafish for developing a new animal model for OPIDN was explored by analyzing the effect of two prototypic neuropathic OPs on PV-NTE activity and PCs, LPCs and GPC profiles in zebrafish brain, as well as their ability to induce distal axonopathy at the spinal cord level and delayed impairment of the motor output. The level of PVase activity and the proportion of NTE inside the pool of esterases were determined using selective inhibition by paraoxon and mipafox, as they are usually determined in hen brain the usual model for testing OPIDN^8^ and in other tissues/species^34,50^.

Although the development of OPIDN in zebrafish has not been tested, zebrafish models of other motor neuron diseases have already been developed^37–40^. As sensitivity to OP-induced delayed neuropathy in other species has been reported to increase with age^1,51^, we chose to use adult zebrafish in this study instead of embryos or early larvae. We selected two neuropathic compounds, CBDP and DFP, to assess their effect on the NTE activity in the brain. CBDP is the neuropathic metabolite of TOCP and is formed *in vivo* by two consecutive reactions: (1) liver microsomal cytochrome P450-catalyzed oxidation and (2) serum albumin-catalyzed cyclization of the oxidation product^52,53^. We used CBDP directly instead of the parental compound, TOCP, because zebrafish have no albumin^54^, and we could not predict whether TOCP would be metabolically activated in zebrafish. DFP is a neurotoxin analog of the chemical warfare agents sarin and soman and has a potent inhibitory effect on NTE^53,55^. The proportion of PV-NTE activity was 6% of total PVase activity determined in this study in zebrafish brain, and the absolute value in term of activity per gram tissue, is approximately 10-fold lower than that reported for hen brain^56^ but was nonetheless within the same unit range and similar to that observed in hen peripheral nerve, and much lower than that in adrenal medulla chromaffin cells as that is the tissue with higher NTE content that has been reported^57^. As described in mammalian models and hens, zebrafish NTE inhibition by both compounds was dose- and time-dependent. However, in zebrafish, both compounds showed a stronger inhibitory potency for NTE than for AChE. The comparison of efficiency of OP esters against AChE and NTE has been validated as a predictive tool to determine whether a compound will be neuropathic at doses below or above LD_50_ values^8^ and the basis of a proposal for *in vitro* testing^58^. If an OP compound is a substantially more potent inhibitor of AChE than of NTE, cholinergic toxicity could result in lethality, and no neuropathic outcome would occur. However, if the inhibitory potency of the OP compound for NTE is higher or similar to for AChE, cholinergic toxicity will be mild at concentrations inducing enough inhibition of NTE for initiating the delayed neuropathy and the compounds will be labeled as “neuropathic”. As reported for hens, rodents and humans^59–61^, in this study, both tested compounds exhibited properties of neuropathic agents since at non-cholinergic lethal doses both were able to inhibit over 70% of the NTE activity for more than 24 h, which is one of the known requirements for OPIDN induction in humans chickens, cats, and farm animals^62^. Interestingly, in this study the selectivity of DFP for zebrafish brain NTE relative to that of AChE was very high, as concentrations of this compounds inhibiting over 70% of NTE activity had only a mild and not significant inhibitory effect on AChE activity (9.3–26.8%). Similarly, DFP has been reported to be approximately 7.5-fold more efficient *in vitro* inhibitor of NTE than AChE in mouse brain^63^. Not only must OP compounds irreversibly and significantly inhibit NTE (>70%) within 1–2 days to induce OPIDN, but the interaction between the OP compound and NTE must be causing a modification referred to as aging. NTE aging is operatively defined as the loss of the reactivation capacity of the protein by nucleophilic agents, such as KF^64^ and molecularly is due to a dealkylation of one of the alkylgroup in the organophosphorylated protein. In this study, aging for zebrafish NTE inhibited *in vivo* by CBDP and DFP was shown for the first time. Both OPs are well known NTE aging agents in usual OPIDN model species including humans^14,18,58,64–67^. Zebrafish NTE brain enzymes inhibited by OPs exhibited a time-dependent loss of the ability to be reactivated by nucleophiles in zebrafish brain tissue. With DFP, it is well established, that NTE aging in hen brain tissue happens within minutes^64,65^ to a maximum of 13% (*in vitro*). Here we observed that 1 h following *in vivo* exposure to DPF, zebrafish brain NTE activity recovered 60.02% (40% of aged protein) after incubation with the reactivation agent, on the other hand, at 6 and 12 h post exposure, reactivation decreased to similar levels reported in hens^64,65^. CBDP, was a more potent aging agent than DPF, and varied much less across time (between 25.77 and 12,62% vs 60.02 and 14.45%, respectively). However, although CBDP, as previously mention, is very well known for its implication in the development of OPIDN, hence inhibition and aging of NTE, there is very little scientific reports that document biochemically its aging activity. Only one biochemical report could be found regarding NTE aging by CBDP^14^ where OPIDN was induced in hens using a single dose of tri-o-cresyl phosphate (TOCP; parent compound of CBDP) and reported 13.2; 17.16 and 12.62% of enzyme aging in brain tissue, 4 hours, and 4 and 14 days post exposure, respectively. CBDP exposed zebrafish, exhibited NTE aging within the first 24 h ranging from 74.23 to 87.38%, over 5 fold higher than that described in hens^14^. Such a large discrepancy is very difficult to explain, on the other hand, like in hens, aging levels in exposed zebrafish varied little across time.

The effect of neuropathic OPs on PCs, LPCs and GPC profiles remains controversial. Whereas some reports in drosophila and mice indicate an increase in the level of PC by exposure to neuropathic OPs^13,68^, other studies in mice and hens reported no effect on PC and LPC homeostasis^14,15^. Currently, only one study has addressed the effect of neuropathic OPs on the PCs, LPCs and GPC profiles^12^. In that study, authors analyzed by HLC-ESI-MS/MS the phospholipidome in the endoplasmic reticulum (ER) fraction of the spinal cord of hens 48 h after animals were dosed with TOCP and PMSF + TOCP, finding a significant increase in some PCs and LPCs, and a decrease in GPC. Although we have found also a significant increase in some LPCs in the brain of zebrafish 48-h after treatment with DFP, no effects were detected in the PCs or GPC levels. Zebrafish NTE/PNPLA6 protein shares 73% identity with human PNPLA6, and contains an acyltransferase/acyl hydrolase/lysophospholipase domain (IPR016035) with 86.2% identity with the human one^43^. Moreover, it has been demonstrated in human and sensitive species that this catalytic domain is involved in the PV-NTE activity and LPB and LysoPLA activities exhibited by NTE/PLNA6 protein and good correlation has been found in the sensitivity of PV-NTE and LysoPLA activities in mice exposed to different neuropathic OPs^10^. Thus, although LysoPLA and PLB activities have not been analyzed in this study, the high degree of inhibition of PV-NTE activity obtained for both compounds in zebrafish strongly suggest that the phospholipase activities of the enzyme are also inhibited. In the present study, no changes in the PCs and LPCs profiles were found in the brain of adult zebrafish exposed to 150 mg/kg CBDP for 6 and 48 h, although PV-NTE inhibition was 96.9% and 74.3%, respectively. Similarly, no changes in the PCs profile were found in the brain of adult zebrafish exposed to 300 mg/kg DFP for 6 and 48 h, although PV-NTE inhibition was 88.0% and 85.8%, respectively. These results suggest that in zebrafish there are other enzymes involved in the PC metabolism able to compensate, at least partially, the inhibitory effect of neuropathic OPs on PNPLA6.

The role of NTE protein and its encoding gene in neurodevelopment has been associated to alteration of lipids homeostasis and membrane formation either in mouse cell^48^ or in human derived cells^44^ but is was also demonstrated that this role is not due to the catalytic PV-NTE activity but any other unknown function of the protein and gene in the differentiation, as it was observed that blocking the gene by iRNA produces strong effect on nervous system differentiation. However, these effects were not observed by simply NTE by neuropathic compound as mipafox in an *in vitro* model with human derived cells^69^ and in mice^47^.

When the motor function outcome was analyzed in zebrafish 1–3 weeks after a single dose of 150 mg/kg CBDP or 300 mg/kg DFP, no significant differences were found in the distance moved by the fish at any time. The OFT has been used to evaluate hindlimb dysfunction in both nestin-cre:NTEfl/fl mice with NTE-deficient neural tissues as well as in 57BL/6 J mice acutely dosed with a neuropathic OP. Whereas some changes in the open-field performance were found in the nestin-cre:NTEfl/fl mice, no clinical signs of hindlimb dysfunction were detected using this test in the mice acutely dosed with the neuropathic OP^13^. The fact that no effects on the motor outcome are evident in the OFT indicates that the generation of the simple swim pattern is not altered in the treated fish.

It has been reported that in some low sensitive species, neuropathic OPs may achieve NTE inhibition and aging without any apparent clinical effect. Interestingly, in these species high NTE inhibition correlated with distal axonopathy, although no clinical effects were evoked^13,70^. However, when the spinal cord of adult zebrafish treated with neuropathic doses of CBDP and DFP for 1–3 weeks was examined for presence of histopathology, no degenerative changes associated with neurotoxic compounds were found. One potential explanation for the absence of morphological alterations in the spinal cord at light microscopy level in zebrafish would be if OPIDN development requires the disruption of the phospholipid homeostasis. However, the fact that the homeostasis of PC and LPC was not disrupted during TOCP induced OPIDN in hens^14^ suggest that other mechanisms could be involved in the unsusceptibility of zebrafish to develop OPIDN. In humans and other sensitive species, OPIDN results from the Wallerian degeneration of the longest axons in both the peripheral and central nervous systems. Whereas peripheral axons can regenerate, regeneration of central axons does not occur in mammals^71^. Thus, patients with mild cases of OPIDN will show clinical improvement and recovery as peripheral nerves regenerate. However, severe cases of OPIDN that involve damage to the CNS develop a persistent spasticity^1^. In contrast to mammals, functional regeneration of the CNS occurs in teleost fish^72–75^. Thus, adult zebrafish is able to recover locomotor function, although the recovery at histological level is only partial. The absence of degenerative changes associated with OPIDN in the spinal cord of the adult zebrafish exposed could be related also by ability of this species for axonal regrowth after injury. However, further studies would be necessary to dissect the mechanisms involved in the resistance of zebrafish to develop OPIDN.

## Material and Methods

### Animals and housing

Adult wild-type zebrafish were raised and maintained in the Research and Development Centre of the Spanish Research Council facilities (registration number B9900083) according to standard protocols^76^. All procedures were conducted in accordance with the institutional guidelines under a license from the local government (DAMM 8871, 7964) and were approved by the Institutional Animal Care and Use Committee at the Spanish Research Council.

### Chemicals

Diisopropylphosphorofluoridate (DFP - CAS: 55-91-4), paraoxon (CAS: 311-45-5), benzenesulfonyl fluorid (BFS, CAS:368-43-4), chloroform and butylated hydroxytoluene (BHT) were purchased from Sigma-Aldrich (St. Louis, MO, USA). PV and mipafox were purchased from AccuStandard (New Haven, CT, USA) and Chembridge (San Diego, CA, USA), respectively.

### Synthesis of 2-(2-cresyl)-4H-1-3-2-benzodioxaphosphorin-2-oxide (CBDP)

High-performance liquid chromatography (HPLC)-grade water and methanol were purchased from Fisher Scientific (USA). Internal standards for lipidomics were purchased from Avanti Polar Lipids. Finally, 2-(2-cresyl)-4H-1-3-2-benzodioxaphosphorin-2-oxide (also called cresyl saligenin phosphate) was synthesized in-house using an established two-step procedure for similar phosphate triesters. In the first step, cresyl phosphorodichloridate was obtained by treatment of o-cresol (1 equiv) with phosphoryl chloride (POCl3, 1 equiv) in the presence of anhydrous triethylamine (NEt3, 1 equiv) using dry diethyl ether (Et2O) as the solvent at −78 °C. The precipitate was filtered, and the organic solvent was concentrated to obtain the desired intermediate as an oil. In the second step, the obtained cresyl phosphorodichloridate reacted with 2-hydroxybenzyl alcohol (1 equiv) using dry methylene chloride (CH2Cl2) as a solvent in presence of anhydrous NEt3 (3 equiv) at 0 °C and then at room temperature for 2 h. Formation of the desired compound was monitored by TLC. After this period, the organic phase was washed with water, and the resulting solvent was removed under reduced pressure to yield a residue containing the desired cyclic phosphate triester. This compound was purified by flash SiO2 column chromatography using a stepwise gradient with hexanes/AcOEt (0–30%, 1% each SiO2 solvent volume). The overall yield was 60%.

^1^H NMR (400 MHz, CDCl _3_) δ 7.40–7.25 (m, 2 H), 7.22–7.05 (m, 6 H), 5.55–5.40 (m, 2 H), 2.21 (s, 3 H).

^13^C NMR (101 MHz, CDCl_3_) δ 150.0 (d, *J* = 7.0 Hz), 148.7 (d, *J* = 8.0 Hz), 131.52, 129.9 (d, *J* = 2.0 Hz), 129.1 (d, *J* = 6.5 Hz), 127.2 (d, *J* = 1.5 Hz), 125.5 (d, *J* = 1 Hz), 125.4 (d, *J* = 1 Hz), 124.6, 120.5 (d, *J* = 10.0 Hz), 119.7 (d, *J* = 2.5 Hz), 118.8 (d, *J* = 9 Hz), 69.2 (d, *J* = 7.5 Hz), 16.1.

^31^P NMR (162 MHz, CDCl_3_) δ −15.8.

### Experimental procedure

Only male adult zebrafish were used for the exposure experiments. DFP and CBDP stock solutions were prepared in pure corn oil (sigma) and concentrations were adjusted to inject approximately no more than 1% of body weight. After cold water anesthesia (12 °C) of the fish, body length (BL) and weight were measured before the toxicant or the carrier was administered intraperitoneally (ip)^77^. Then, range-finding tests for both CBDP and DFP were carried out, with an initial selected endpoint of survival after 24 h of exposure. Dose-response, time-course and motor function outcome experiments were performed in duplicate. At all the selected times (1 h, 3 h, 6 h, 24 h, 48 h, 1 wk and 3 wk post-exposure), fish were euthanized by inducing hypothermic shock in ice-chilled water (2° to 4 °C), and the brains were immediately collected and stored at −80 °C for PV-NTE and AChE activities analyses.

### Analysis of NTE activity in zebrafish brain

The PV-NTE activity protocol for brain tissues of adult zebrafish was adapted from Johnson (1977)^8^ to low volume assessment of NTE activity, where the reaction volume was 400 µL. Brain samples were homogenized in homogenization buffer [ice cold 50 mM Tris-HCl (pH 8.0) plus 1 mM EDTA buffer] to a 12 mg/mL (tissue:volume) proportion, using TissueLyser LT (Qiagen) with stainless steel beads. Activity was determined at 37 °C for 20 min by incubating brain homogenates with phenyl valerate (PV) (0.5 mM final concentration) prepared in homogenization buffer containing 0.03% Triton X-100. The reaction was stopped with 200 µL 1.23 mM 4-aminoantipyrine prepared in 2% sodium dodecyl sulfate solution. The phenol formed from PV hydrolysis was measured by the addition of 50 µL of 6.5 mg/mL potassium ferricyanide prepared in 50 mM Tris-HCl (pH 8.0) and incubation for 5 min to allow color development followed by absorbance measurement at 490 nm using a microplate reader (Synergy 2 multi-mode, ®BioTek Instruments Inc., Winooski, VT, USA).

PV-NTE activity, defined as the paraoxon-resistant and mipafox-sensitive activity, was then calculated by measuring the difference in absorbance between samples that were pre-incubated for 20 min at 37 °C with either 15 µM paraoxon (prepared in acetone) or 15 µM paraoxon plus 100 µM mipafox (prepared in 10 mM Tris-Citrate buffer pH 6.0) prior to the addition of PV.

### Measurement of NTE reactivation and aging

The following method has been previously validated for the zebrafish brain tissue (data not shown). Homogenates of brain tissue (12 mg/mL) from fish collected 1, 6, 12 and 24 h after exposure were used to measure NTE aging. Samples were analyzed in pairs of two (set A and set B), where 400 mM of potassium chloride (KCl) and 400 mM of potassium fluoride (KF) was added to tube A and B, respectively, to a final concentration of 200 mM (1:1). KF acts as a reactivation reagent while KCl serves as an ion strength control. Both reagents were prepared in 50 mM Tris/citrate buffer pH 5.2 containing 1 mM EDTA. Samples were then incubated for 20 min at 37 °C, after which they were transferred to ice to slow the reactivation process and then centrifuged at 2–4 °C at 20000 × g for 40 min to eliminate the chloride and fluoride. During the validate process, two reactivation time periods with KF was tested in treated samples, 20 and 60 min, no difference between enzyme activities was observed, thus we selected the shortest time period for our analysis. The resulting pellet was then resuspended in homogenization buffer to a tissue concentration of 12 mg/mL. PV-NTE activity was then measured as described above using 100 μM of benzenesulfonyl fluorid (BFS), prepared in acetone (lowest dose where PV activity was no longer inhibited), instead of paraoxon^58,65^. Results were expressed in percentages with respect to control subjected to the same assay conditions. No difference was found in activities treated with KF and KCl from control samples. Reactivation of PV-NTE activity was calculated as follows:

$${\rm{Reactivation}}={\rm{100}}\times [( \% \,{{\rm{NTE}}}_{{\rm{KF}}}- \% \,{{\rm{NTE}}}_{{\rm{KCl}}})/({\rm{100}}- \% \,{{\rm{NTE}}}_{{\rm{KCl}}})]$$

### Determination of AChE activity

AChE activity was determined in the same brain tissues used for time course PV-NTE assessment. The remaining homogenate sample after NTE analysis was centrifuged at 10,000 × g (4 °C) for 10 min, and the resulting supernatant was collected and stored at −80 °C for AChE determination. AChE activity was determined by adding 2 mM acetylthiocholine and 0.33 mM 5,5′-dithiobis-2-nitrobenzoic acid (DTNB) to the sample. The formation of the product resulting from the reaction between thiocholine and DTNB at 25 °C was monitored for 15 min at 405 nm by a microplate reader. All samples were assayed in triplicate. The results were normalized to the total protein content determined with the Bradford method^78^. The final results are expressed in nmol min^−1^ mg protein^−1^ using the extinction coefficient 13.6 × 10^3^ M cm^−1^.

### Lipidomic analysis

Each zebrafish brain sample was homogenized in 50 mM Tris buffer (pH 8.0) containing 1 mM EDTA and 0.01% BHT at a concentration of 12 mg/mL. Determination of protein with the Bradford method^78^ was carried out prior to the analysis for final normalization of the obtained results. Lipid extraction from zebrafish brain was performed with similar extraction conditions as described by Christie *et al*.^79^, with minor modifications. Briefly, 120 μL of the homogenized sample was mixed with chloroform:methanol (2:1, v/v). Internal standards (200 pmol) were also added. Samples were shaken with a vortex and extracted in an ultrasonic bath. Afterwards, samples were heated at 48 °C overnight and dried under N2 the next day. Lipid extracts were reconstituted in 500 μL methanol and dried under N2 again. The obtained extract stored at −80 °C until the analysis. For its analysis, samples were solubilized in 150 μL methanol, centrifuged at 10,000 rpm for 10 min. The supernatant was transferred to a new micro vial and analysed.

Lipidomic instrumental analyses were performed with an Acquity UHPLC system (Waters, USA) coupled to a Waters/LCT Premier XE time-of-flight (TOF) analyzer operated in positive and negative electro spray ionization (ESI) mode. The analytical separation was performed with an Acquity UPLC BEH C8 column (1.7 mm particle size, 10 × 2.1 mm, Waters, Ireland) at 30 °C and a flow rate of 0.3 mL/min. Chromatographic conditions and MS parameters have been previously reported^80^. Fullscan spectra from 50 to 1,800 Da were acquired, and individual spectra were summed to produce data points each of 0.2 sec. Mass accuracy at a resolving power of 10,000 and reproducibility were maintained by using an independent reference spray (Lock Spray Waters). Mobile phases used were A, methanol:2 mM ammonium formiate:0.2%formic acid and B, water:2 mM ammonium formiate:0.2% formic acid. Both phosphatidylcholine (PC) and lyso-phosphatidylcholine (LPC) were analyzed under positive ESI (ESI+).

Identification and relative quantification of lipids was carried out using the ion chromatogram obtained for each compound using 0.05 Da windows. Positive identification of lipids was based on the accurate mass measurement, with a minimum mass error (<5 mg/L) respect to the measured m/z ratio of the monoisotopic peak considering possible adducts, its relative retention time and correct isotopic distribution. Home-made databases of lipids and LipidMaps external online database (http://www.lipidmaps.org) were used in order to identify potential lipids. Individual chromatographic peaks of distinct lipid species were isolated from full-scan MS spectra when selecting their theoretical exact masses, extracted from the databases. A list of possible candidates fitting the specific exact mass was generated using formula determination tools (elemental composition search) of MassLynx software (Waters, USA). Relative quantification was done by comparison of peak areas in extracted ion chromatograms between expected lipids and its corresponding internal standards. Obtained relative lipid quantification in each sample was finally normalized with previously measured protein content. A total of 63 lipids were identified as [M+H]+ and quantified by UPLC-TOF ESI-positive mode, distributed in 48 phosphocholine (PC) and 15 lysophosphatidylcholine (LPC). Both types of glycerophospholipids were annotated as <lipid subclass><total fatty acyl chain length>: <total number of unsaturated bonds>.

### Histopathological evaluation

Standard protocols were used for the histopathological analysis by light microscopy. Zebrafish were immediately fixed in Dietrich’s fixative at room temperature. After 1–2 weeks, 0.4–0.5 mm transversal sections of the fish body at the level of the caudal fin were taken, put in histological cassettes and processed in paraffin according to standard techniques. Sections (4 µm) were stained with Haematoxylin and Eosin (H&E) and Luxol Fast Blue and mounted in DPX. The obtained transverse sections of spinal cord were examined under a light microscope in order to evaluate the presence of absence of alterations.

### Open Field Test paradigm (OFT)

The zebrafish OFT was performed according to Gomez-Canela *et al*.^81^. An experimental setup for monitoring and recording 4 fish simultaneously was used. The OFT was performed in four circular trunked conical white plastic tanks (testing tanks; 22.5 cm lower diameter ×25.0 cm upper diameter ×26.0 cm height) containing 5 L of fish water at 28 °C. Following the exposure to the carrier and CBDP for selected times, fish were individually transferred to the testing tanks. The first 6 min of the trial were video-recorded (MPEG1 format, 30 fps at 640 × 480) with a video surveillance software (Security Monitor Pro; DeskShare Inc., Plainview, NY, USA) linked to USB 2.0 web-cameras (Microsoft LifeCam Studio; Microsoft Corporation, Redmond, WA, USA) placed on the top of the testing tanks. Two anti-flicker LED tubes (TUT8-ST28-NFL; AS de LED®, Valencia, Spain) mounted on both sides of the test tanks provided uniform illumination for the video-recording. The behavioral endpoint selected to determine motor outcome function was the total distance traveled, as this endpoint reflects general motor/neurological phenotypes^82,83^. Thus, after the recording was complete, the videos were analyzed by Ethovision XT 11.5, and the total distance traveled (m) was determined.

### Data analyses

Statistical analysis of NTE activity and behavioral data was performed using SPSS software v22 (IBM, USA). The data are presented as the mean ± SEM unless stated otherwise. Pairwise statistical significance was determined with Student’s *t*-test, one-way ANOVA or Mann-Whitney rank sum test as appropriate. The results were considered significant at p < 0.05 unless otherwise indicated.

Lipidomic data analysis was performed by using two different approaches. In the first approach, using different packages in the R environment^84^, significant changes in lipid composition were tested by two-way ANOVA plus Benjamini-Hochberg *post-hoc* (fdr) test using the stats package. Treatment- and exposure time-related variations of individual lipids were analyzed by ANOVA plus Tukey’s B test using the packages *foreign*, *agricolae*, and *multcomp*. Heatmaps and hyranchical clustering were performed using the heatmap.2 function in *gplots*.

In the second approach, lipidomic data were analyzed by using ANOVA-Simultaneous Component Analysis (ASCA), a multivariate data analysis approach that combines the statistical advantages of ANOVA to separate the variance sources, and the advantages of Principal Component Analysis (PCA) for eliminating covariation among variables and explain maximum variance^85^. In this study, ASCA was applied to an augmented well-balanced data matrix (Supplemental Fig. 6) by using PLS Toolbox 7.8 (eigenvector Research Inc., Wenatche, WA, USA) working in a MATLAB 8.3.0 R2014a environment (The Math Works, Natick, MA, USA). The effects of two categorical factors, the treatment and the exposure time, besides their interactions, were studied. Statistical significances of the two factors and interaction were evaluated by a permutation test, using 10,000 permutations.

## Electronic supplementary material

Supporting Information

Supplementary Dataset1
