# Supplementary material for: Analysis of the neurotoxic effects of neuropathic organophosphorus compounds in adult zebrafish
Source: Sci Rep. 2018 Mar 19;8:4844. doi: 10.1038/s41598-018-22977-4 (PMC5859099; doi:10.1038/s41598-018-22977-4)
Supplement: Supplementary file 1 — Supporting Information [file 41598_2018_22977_MOESM1_ESM.pdf]

Supporting Information (SI)

"Analysis of the neurotoxic effects of neuropathic organophosphorus compounds in adult zebrafish"

Melissa Faria, Inmaculada Fuertes, Eva Prats, Jose Luis Abad, Francesc Padrós, Cristian Gomez-Canela, Josefina Casas, Jorge Estevez, Eugenio Vilanova, Benjamin Piña, Demetrio Raldúa

***Corresponding author:** drpqam@cid.csic.es. Tel: +34-934006100

Title for HTML: Supporting Information

Description: Supplemental Figures 1-6, Supplemental Tables 1-3

Title for HTML: Supplemental Dataset 1

Description: **Concentration of 47 PCs, 15 LPCs and GPC in the brain of adult zebrafish 6 h, 48 h, 1 wk and 3 wk after the injection of the vehicle (corn oil; control group), CBDP (150 mg/kg bw; CBDP group) or DFP (300 mg/kg bw; DFP group). Results are expressed as pmol/mg protein and as pmol/mg brain w.w.**

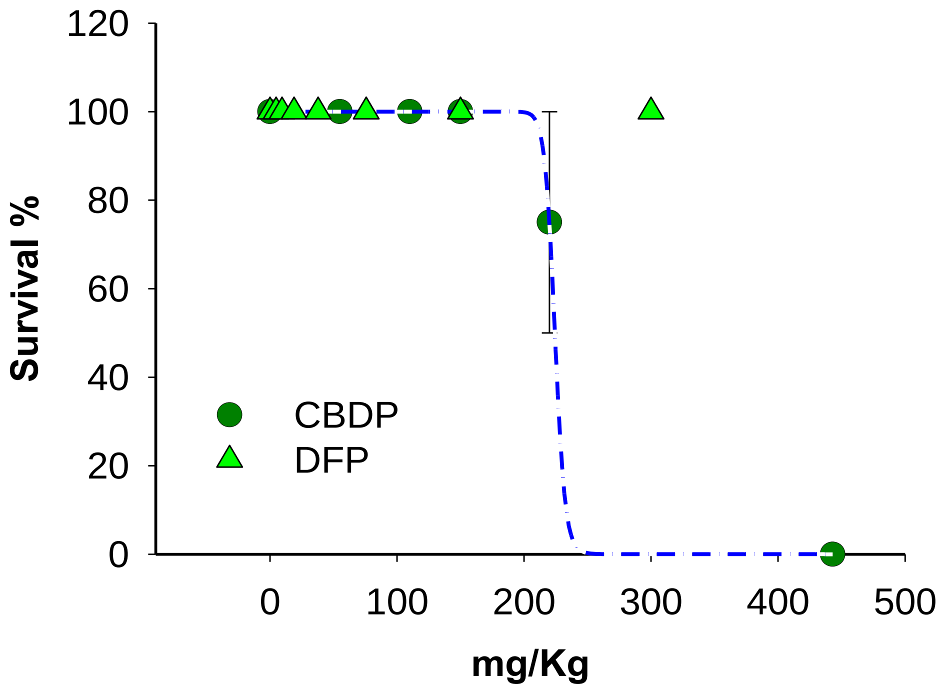

Supplemental Figure 1. Dose-response analysis of the effect of CBDP (150 mg/kg bw, ip; dark green dots) and DFP (300 mg/kg bw, ip; light green triangles) on the survival of adult zebrafish 24 h after exposure. N=20 for each compound, data from two independent experiments. The results are presented as % relative to the control.

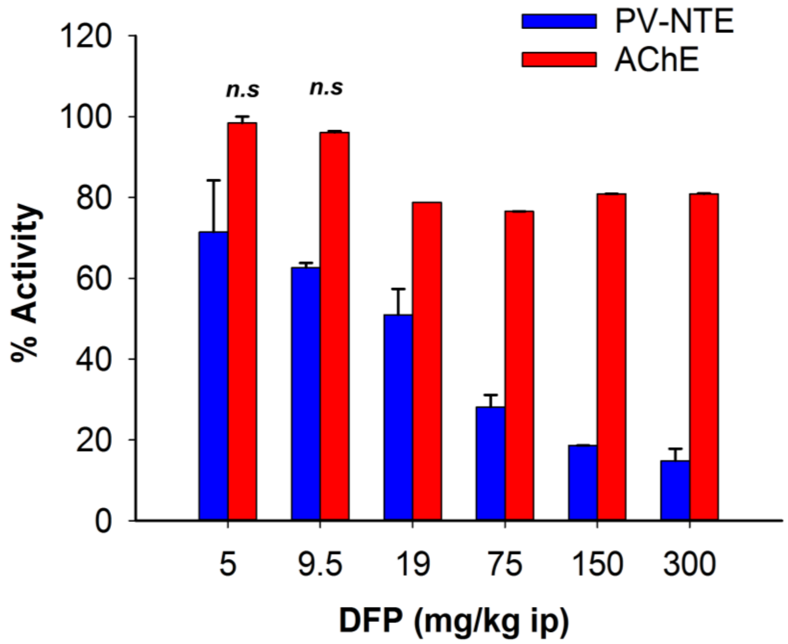

Supplemental Figure 2. Zebrafish brain PV-NTE (blue bars) and AChE (red bars) activities measured 24 hours after a single i.p. dose of DFP. The residual activity is shown as % relative to the control (mean value ± SEM). All results, with the exception of those indicated with n.s. (not significant), were significantly different from the controls.

a

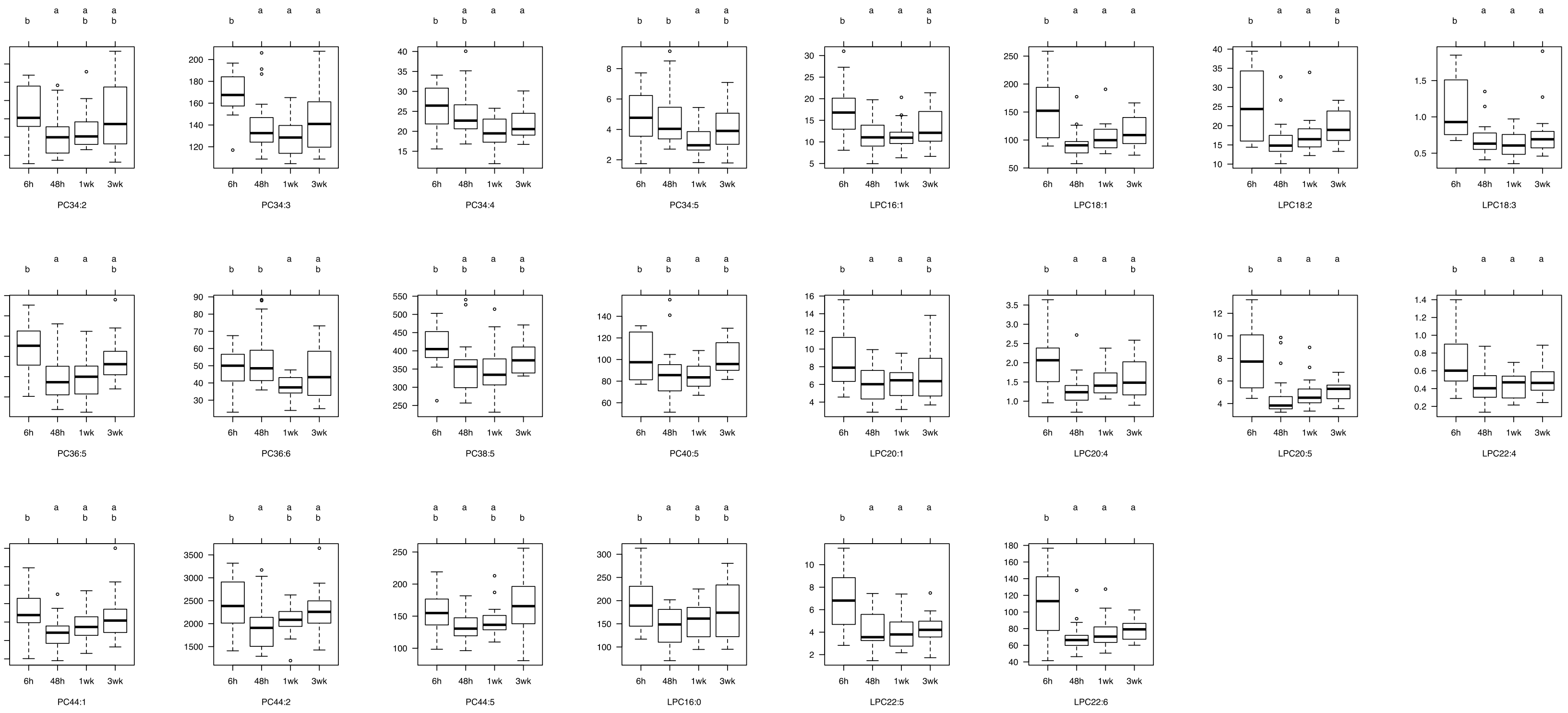
PC34:2
PC34:3
PC34:4
PC34:5
LPC16:1
LPC18:1
LPC18:2
LPC18:3
PC36:5
PC36:6
PC38:5
PC40:5
LPC20:1
LPC20:4
LPC20:5
LPC22:4
PC44:1
PC44:2
PC44:5
LPC16:0
LPC22:5
LPC22:6
6h
48h
1wk
3wk

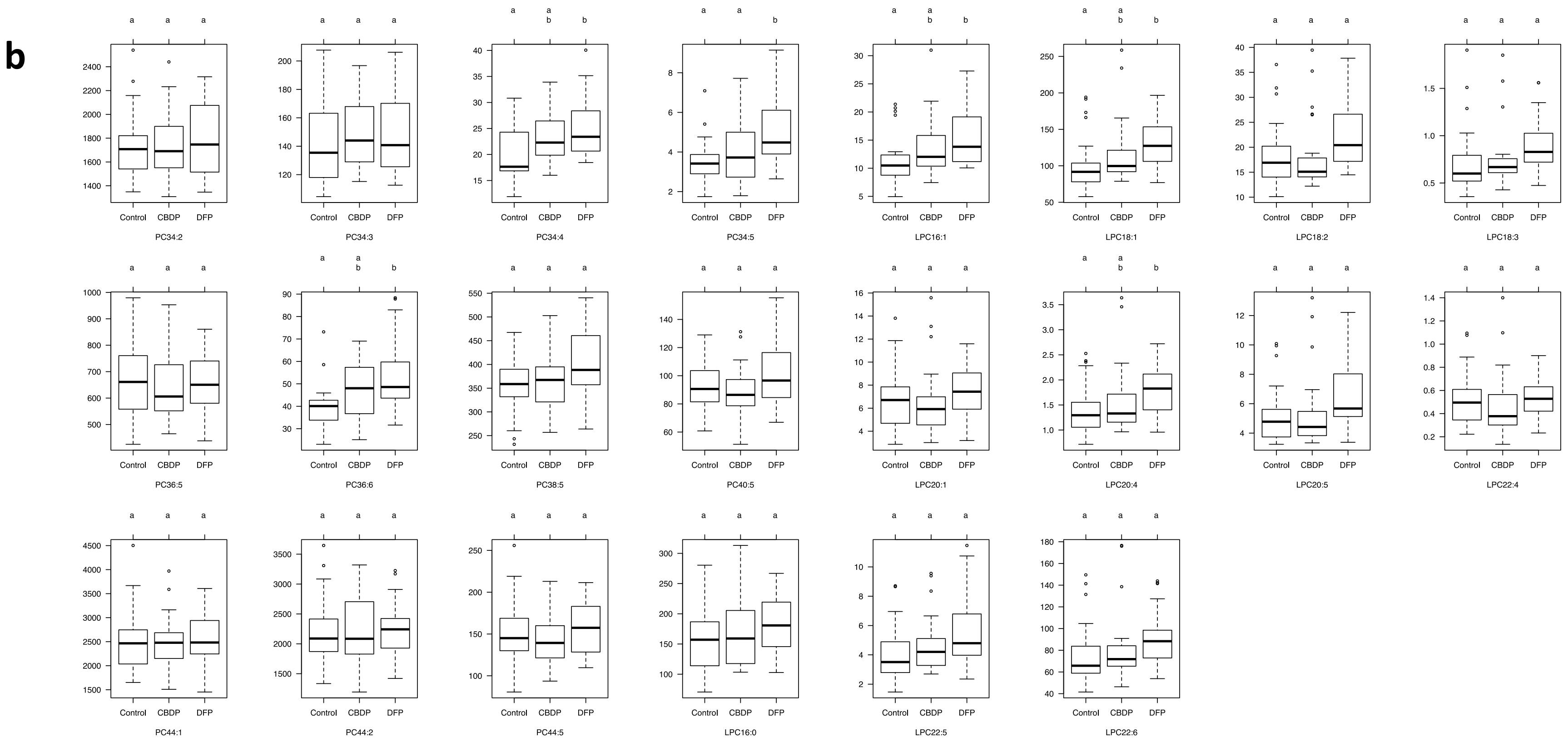

Supplemental Figure 3. Temporal- (a) and treatment-dependent (b) variations on phospholipid content in zebrafish brains from control, CBDP (150 mg/kg bw, ip) and DFP (300 mg/kg bw, ip) groups. The graphs represent phospholipid concentrations per mg of total protein. Mean values are indicated by a thick line, boxes cover second and third quartiles, and whiskers indicate the total distribution, except for outliers (circles). Different letters at the top of the graphs indicate

significant differences (P<0.05) between responses (treatment and time periods) following one way ANOVA and Tukey's *post hoc* comparison test.

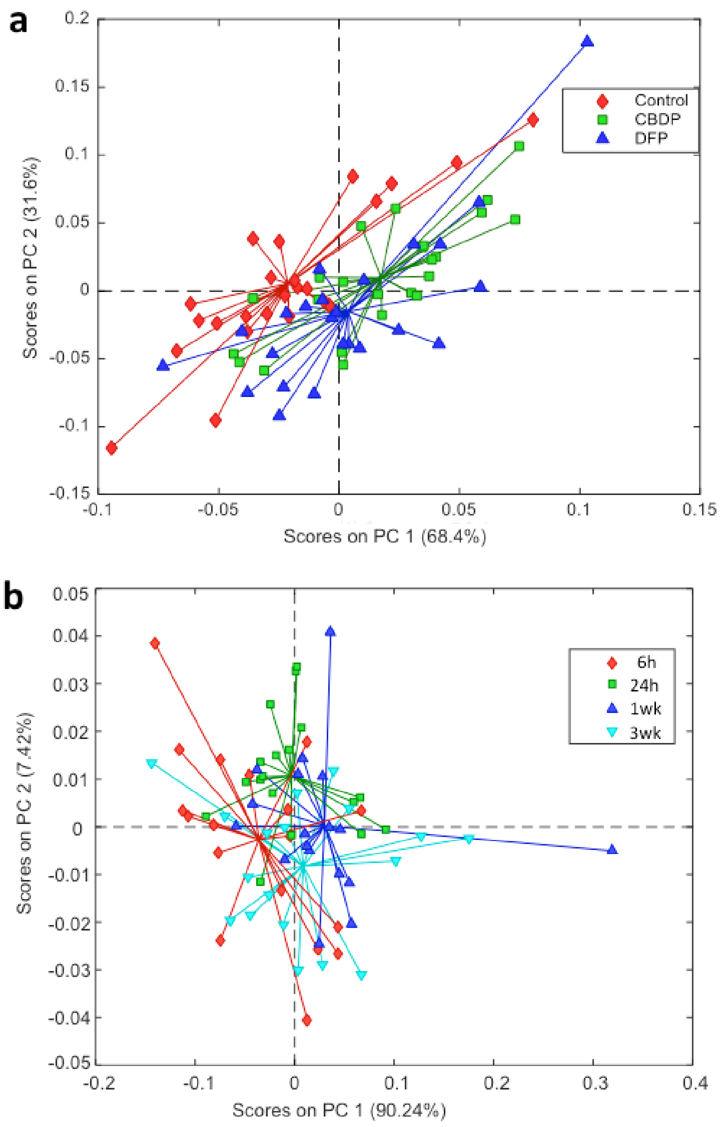

Supplemental Figure 4. ASCA results of on the phospholipid content in zebrafish brains from control, CBDP (150 mg/kg bw, ip) and DFP (300 mg/kg bw, ip) groups. (a) SCA scores plot of the "treatment" factor matrix. Color symbols indicate the different samples studied: red diamonds are controls, green squares are the zebrafish samples exposed to CBDP and blue triangles are the zebrafish samples exposed to DFP. (b) SCA scores plot of the "exposed time" factor matrix. Color symbols indicate the different samples studied: red diamonds are 6 hours exposed samples, green squares are 48 hours

exposed samples, up blue triangles are 1 week samples and down cyan triangle are 3 week exposed samples.

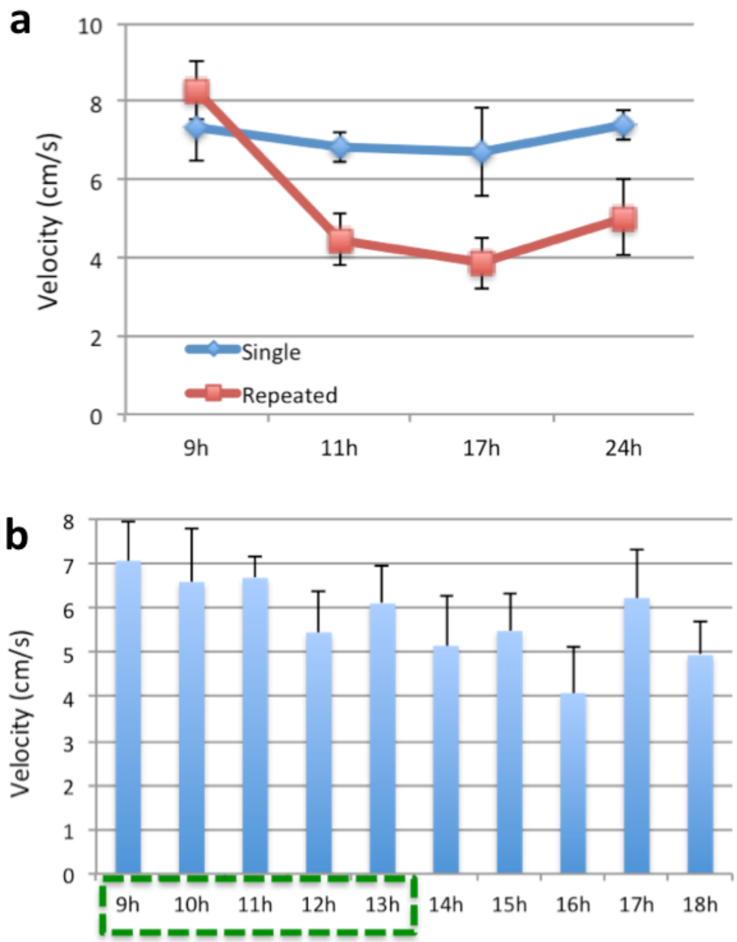

Supplemental Figure 5. Assessing the effects of habituation and circadian rhythms on the performance of control zebrafish in the Open Field Test (OFT). (**a**) When the OFT was performed on the same animal at different times, a significant decrease in velocity was found; “Single”: the velocity was measured on different animals at each selected time, avoiding in this way the potential effect of habituation; “Repeated”: the velocity was measured on the same animals at all selected time, assessing in this condition the potential effect of habituation. (**b**) when OFT was performed at different times of the day, from 09:00 to 18:00 h, the period with the highest stability was from 09:00 to 13:00 h. Note that

a different group of fish was used each time to avoid the confounding factor of habituation.

Data presented as mean ± SEM.

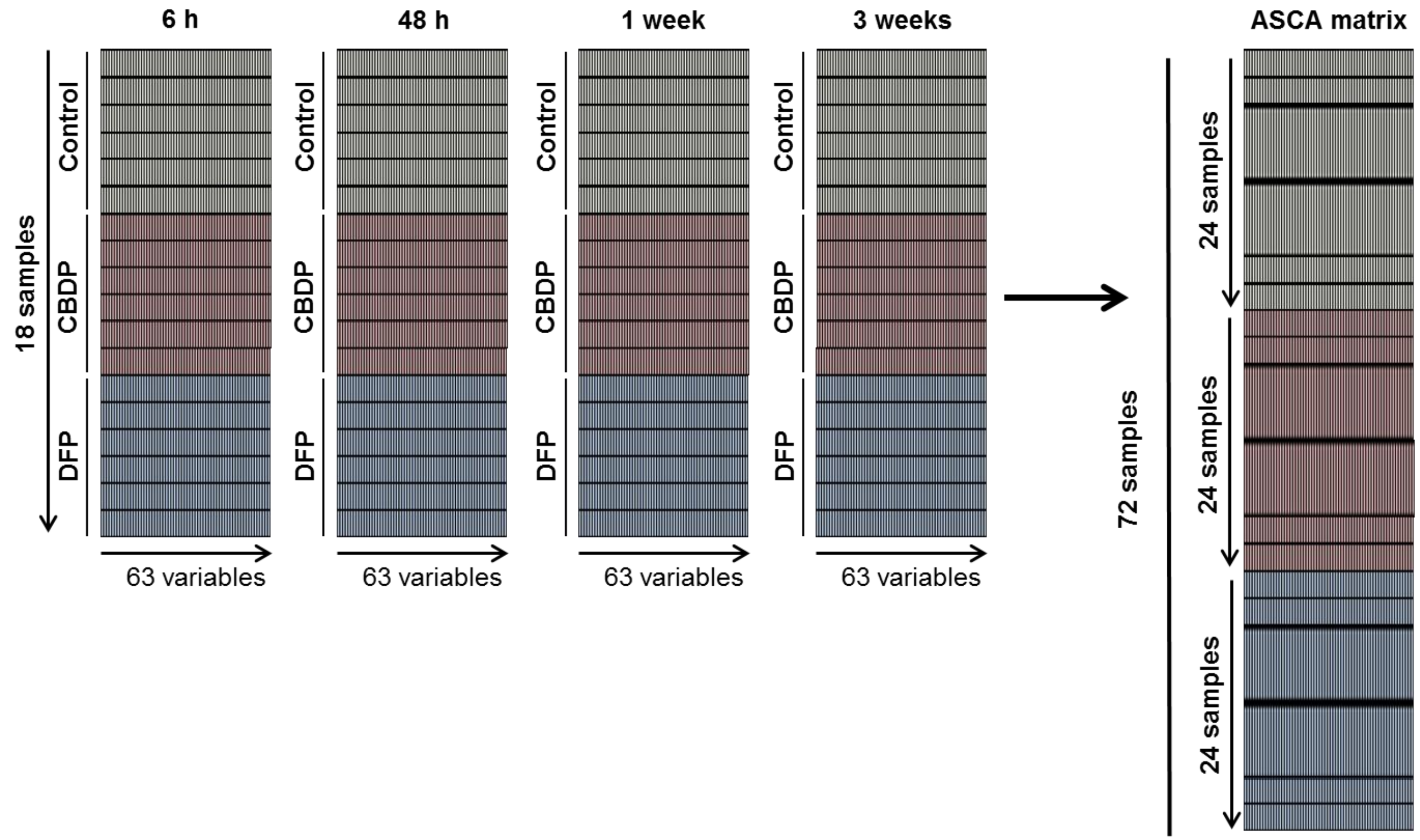

Supplemental Figure 6. Structure of the experimental data sets arranged for ASCA analyses. Each rectangle represents a zebrafish sample which is divided in 63 columns representing the lipids identified. Data sets at each treatment are shown in different colors [green: controls, red: CBDP (150 mg/kg bw, ip) and blued: DFP (300 mg/kg bw, ip)]. Data sets from control and treated zebrafish samples in the four exposure times are further arranged into an augmented data matrix, as indicated in the right-hand side of the figure.

Supplemental Table 1: Time-course of total PVase (A), paraoxon-resistant (B), (paraoxon+mipafox)-resistant (C) and PV-NTE (B-C) activities in brains from control, CBDP (150 mg/kg bw, ip), and DFP (300 mg/kg bw, ip) exposed adult zebrafish. Results are expressed as mean ± SEM.

		PVase activity nmol min^{-1} gww^{-1} after preincubation with:							
		**A**		**B**		**C**			
Treatment	Time after exposure (h)	Buffer	*P* value	paraoxon	*P* value	paraoxon+mipafox	*P* value	PV-NTE (**B-C**)	*P* value
	1	3915,7 ± 622,5		528,8 ± 62,6		309,9 ± 32,8		218,9 ± 35,5	
	3	3097,9 ± 419,7		400,8 ± 21,2		238,4 ± 15,4		162,4 ± 13,5	
	6	3584,4 ± 480,8		614,6 ± 107,1		305,3 ± 34,7		309,3 ± 79,2	
control	24	3540 ± 677,4		497,01 ± 72,2		284,8 ± 36,3		212,2 ± 40,9	
	48	2486,1 ± 478,2		373,9 ± 45,5		237,9 ± 22,7		136,0 ± 25,5	
	168	3862,0 ± 213,4		525,1 ± 58,23		329,4 ± 35,8		195,6 ± 26,6	
	504	3626,3 ± 454,3		613,6 ± 47,92		397,4 ± 39,8		216,2 ± 37,3	
	1	605,8 ± 64,6	**	228,5 ± 32,9	**	203,4 ± 23,0	*	25,2 ± 13,9	**
	3	310,5 ± 43,9	**	165,2 ± 20,9	**	152,4 ± 19,5	*	12,8 ± 3,4	**
	6	400,2 ± 53,7	**	222,4 ± 22,7	**	213,5 ± 27,0	*	11,0 ± 11,0	**
150mg/kg CBDP	24	422,7 ± 79,5	**	228,2 ± 22,9	**	217,2 ± 27,8	*	11,0 ± 12,2	**
	48	587,9 ± 108,6	**	265,9 ± 26,1	**	225,0 ± 19,3	*	40,8 ± 10,8	**
	168	2795,4 ± 222,3		393,3 ± 22,6		290,0 ± 10,5		103,3 ± 19,0	*
	504	3509,0 ± 202,8		586,9 ± 35,8		354,3 ± 24,1		232,5 ± 38,9	
	1	1019,9 ± 74,9	**	362,3 ± 38,1	**	310,4 ± 26,6		51,9 ± 16,4	**
	3	888,6 ± 67,4	**	236,6 ± 20,0	**	222,6 ± 23,0		14,0 ± 8,3	**
	6	762,9 ± 66,9	**	306,0 ± 30,1	**	294,0 ± 42,3		12,0 ± 15,7	**
300mg/kg DPF	24	658,4 ± 40,2	**	271,5 ± 13,0	**	243,1 ± 11,0		28,4 ± 8,0	**
	48	639,4 ± 22,5	**	258,5 ± 20,6	**	223,3 ± 17,0		36,2 ± 13,3	**
	168	1013,2 ± 77,3	**	420,6 ± 16,3		334,3 ± 13,1		86,3 ± 13,1	**
	504	4233,7 ± 474,7		559,2 ± 35,1		353,9 ± 18,2		205,3 ± 18,2	

* $P<0.05$ and ** $P<0.01$ (Student's *t*-test).

Supplemental Table 2. Time-course inhibition of adult male zebrafish brain PV-NTE and AChE activities, represented as % of activity relative to their respective controls, after exposure to CBDP (150 mg/kg bw, ip) and DFP (300 mg/kg bw, ip). Additionally, the total number of control and treated individuals, total number of independent experiments conducted and *P* values (Student's *t*-test) of each enzyme/time responses are indicated. These data correspond to those plotted in Fig. 1 in the main text.

		150mg/kg **CBDP**				300mg/kg **DFP**			
Enzyme	Time after exposure (h)	Activity % (mean±SEM)	Total sample size control/treated	Independent experiments	*P* value	Activity % (mean±SEM)	Total sample size control/treated	Independent experiments	*P* value
PV-NTE	1	11.49 ± 6.39	9 / 9	3	0.00058 **	23.7 ± 7.48	9 / 10	3	0.00002 **
	3	7.88 ± 2.11	4 / 4	1	0.00028 **	8.64 ± 5.10	4 / 4	1	0.00019 **
	6	3.55 ± 3.56	9 / 8	3	0.00458 **	3.87 ± 5.1	9 / 9	3	0.00017 **
	24	5.18 ± 5.76	9 / 9	3	0.00125 **	13.39 ± 3.7	9 / 9	3	0.00132 **
	48	24.65 ± 6.51	9 / 8	3	0.00825 **	21.86 ± 8.02	9 / 10	3	0.00168 **
	168	52.81 ± 9.73	6 / 6	2	0.00896 **	44.11 ± 6.78	6 / 6	2	0.00202 **
	504	107.54 ± 18.01	6 / 6	2	0.32508	94.97 ± 8.40	6 / 6	2	0.29808
AChE	1	57.93 ± 15.41	3 / 3	1	0.01594 *	88.39 ± 11.36	3 / 4	1	0.05897
	3	29.24 ± 19.67	3 / 3	1	0.00179 **	95.89 ± 13.47	3 / 4	1	0.11932
	6	30.29 ± 13.42	3 / 3	1	0.00101 **	93.93 ± 9.36	3 / 4	1	0.10233
	24	26.70 ± 8.34	3 / 3	1	0.00017 **	89.82 ± 4.29	3 / 4	1	0.00936 **
	48	32.67 ± 0.733	3 / 3	1	0.0001 **	91.18 ± 7.97	3 / 4	1	0.11079
	168	87.50 ± 12.02	6 / 6	1	0.29322	89.42 ± 10.38	6 / 6	1	0.4668
	504	99.63 ± 19.54	6 / 6	1	0.16307	107.84 ± 9.65	6 / 6	1	0.11284

***P* < 0.01 (Student's *t*-test).

Supplemental Table 3. ASCA results of on the phospholipid content in adult zebrafish brains from control, CBDP (150 mg/kg, ip) and DFP (300 mg/kg, ip) groups. Significance and partitioning of the total variance into the individual terms corresponding to factors and interaction.

Factor	Cum EigenVal[a]	Percentage of variation[b]	Significance (*p*-value)
Treatment[c]	3.8e-4	4.2	0.19
Exposure Time[d]	6.4e-4	6.9	0.12
Treatment x ExposureTime	9.4e-4	10.2	0.23
Residuals		78.7	

[a]Cumulative EigenVal; [b]Percentage of variation expressed as sums of squared deviations from the overall mean; [c]Treatment (controls, CBDP and DFP); [d]Exposure Time (6 h, 48 h, 1 wk and 3 wk).
